# Neural Correlates of Attentional Flexibility during Approach and Avoidance Motivation

**DOI:** 10.1371/journal.pone.0127203

**Published:** 2015-05-22

**Authors:** Rebecca D. Calcott, Elliot T. Berkman

**Affiliations:** Department of Psychology, University of Oregon, Eugene, OR, United States of America; Centre de Neuroscience Cognitive, FRANCE

## Abstract

Dynamic, momentary approach or avoidance motivational states have downstream effects on eventual goal success and overall well being, but there is still uncertainty about how those states affect the proximal neurocognitive processes (e.g., attention) that mediate the longer-term effects. Attentional flexibility, or the ability to switch between different attentional foci, is one such neurocognitive process that influences outcomes in the long run. The present study examined how approach and avoidance motivational states affect the neural processes involved in attentional flexibility using fMRI with the aim of determining whether flexibility operates via different neural mechanisms under these different states. Attentional flexibility was operationalized as subjects’ ability to switch between global and local stimulus features. In addition to subjects’ motivational state, the task context was manipulated by varying the ratio of global to local trials in a block in light of recent findings about the moderating role of context on motivation-related differences in attentional flexibility. The neural processes involved in attentional flexibility differ under approach versus avoidance states. First, differences in the preparatory activity in key brain regions suggested that subjects’ preparedness to switch was influenced by motivational state (anterior insula) and the interaction between motivation and context (superior temporal gyrus, inferior parietal lobule). Additionally, we observed motivation-related differences the anterior cingulate cortex during switching. These results provide initial evidence that motivation-induced behavioral changes may arise via different mechanisms in approach versus avoidance motivational states.

## Introduction

The type of motivation that propels a goal has downstream effects on the neurocognitive processes engaged in the pursuit of that goal, the specific behaviors that are enacted in turn, and, ultimately, goal achievement. The two fundamental types of motivation alluded to above are approach and avoidance, or the tendency to move towards or away from a stimulus, respectively [1]. Excessive reliance on avoidance goals can diminish well being (e.g. [2,3]) and may even undermine goal achievement [4]; however, the processes by which avoidance (vs. approach) goals influence global constructs such as well being are unknown. More detailed information about the neurocognitive processes that underlie how people attend and react to their environment may inform knowledge of how motivation affects these longer-term outcomes [3]. Thus, a critical step towards understanding the importance of motivational orientation is to describe precisely how approach and avoidance motivation differ in their effects on the proximal neurocognitive processes that ultimately mediate the effect of motivation on more distal outcomes (e.g., goal attainment). One such process is attentional flexibility, which refers to the ability to shift attention between different objects or levels of focus. This paper extends our previous behavioral work, which established the differential effects of approach and avoidance motivation on attentional flexibility [5], by examining the neural systems that underlie how approach and avoidance motivation affect attentional flexibility.

Previous research exploring the effects of approach and avoidance motivation on flexible cognition has yielded equivocal results. One group of studies evoked approach and avoidance motivation using implicit cues, which are designed to elicit motivational states outside of subjects’ awareness. Friedman & Forster [6] found that implicit approach (vs. avoidance) cues broaden categorization processes and facilitate flexible, creative thinking. By contrast, Koch, Holland and van Knippenberg [7] used embodiment cues to subconsciously evoke motivational states and found that avoidance (vs. approach) led to greater flexibility on a set-shifting task. Other studies have examined approach (or reward) motivation by evoking them more explicitly using performance-dependent rewards and pictures of desirable foods. Motivation to obtain a reward (approach) increases proactive control, which strengthens task maintenance, while at the same time reducing flexibility to respond to unexpected targets [8,9,10]. Additionally, positive states that are high in approach motivation reduce flexibility on an attention shifting task relative to positive states that are low in approach [11]. Although these recent studies did not compare approach and avoidance, they do suggest that strong approach motivation reduces flexible cognition relative to states that are low in approach motivation. Thus, studies that explicitly evoke approach states have tended to find that approach reduces flexibility; however, these results do not agree with those using implicitly evoked approach states. The effects of explicitly-induced avoidance motivation on cognitive flexibility has been less studied; however even within the implicit literature, the findings are not consistent. The mixed evidence regarding the effect of motivation on cognitive flexibility suggests the presence of (unexplored) moderating factors that may explain these discrepant findings.

One potential moderator of the seeming instability of the effects of approach and avoidance on attentional flexibility is variation in task demands. To investigate this possibility, we conducted a series of behavioral studies that added an additional factor beyond previous studies of motivation and attention: task context [5]. Accordingly, our participants completed a global-local composite letter task in which they had to switch between attending global and local features of a stimulus. Critically, the task context was varied by changing the ratio of global to local targets in a block, to create 3 block types: Mostly (75%) global, mostly local, and even (50% global/50% local). Consistent with our hypothesis about the presence of moderating factors, the relationship between motivation and flexibility differed depending on the block context, with approach leading to greater flexibility in mostly global blocks. This effect was driven by faster switching to the rare local targets on mostly global blocks. On the other hand, in two of three experiments, avoidance led to reduced switch costs in even contexts, in which global and local targets were equally likely. This interesting pattern of results suggests that the attentional differences between approach and avoidance may emerge most clearly when task context is also considered. Furthermore, and relevant for the present study, the context-dependent nature of the results suggests that attentional flexibility may be supported by different underlying mechanisms in approach compared to avoidance states, which in turn facilitate performance to different extents depending on the context.

The present studies expand upon this finding of context-specific motivational differences in attentional flexibility by establishing their neural correlates using fMRI. Where behavioral studies have yielded conflicting findings, neuroimaging provides an alternative means to study the differences between approach and avoidance states, and particularly the way those motivational states differentially influence the neural systems that underlie attentional flexibility. For this investigation, we focused on two aspects of attention switching. First, we studied how context and motivation affect the neural systems involved in *preparedness* to switch by examining variation in the neural activation during the period before each trial as a function of the behavioral response that followed. Studying brain activity in the preparatory period in this way may provide important insights about how motivation shifts the balance between flexibility and stability, and is especially relevant here given that the switching in this task is not cued and relatively implicit. Additionally, we investigated which brain regions are relatively more active during the actual shifting of attention across contexts and motivational states.

A broad network involving frontal, parietal, and subcortical brain regions has been implicated in shifting attention between different perceptual dimensions [12]. Furthermore, activation in multiple independent groups of brain regions during the period immediately before a switch appears to give rise to subsequent cognitive flexibility [13]. The four main sources associated with flexibility as identified by Leber et al. [13] using principal components analysis centered around the anterior cingulate cortex (ACC), the dorsal striatum (DS) and subthalamic nucleus, the posterior parietal cortex (PPC), and a group of frontal and parietal regions. The finding of multiple neural sources of flexibility gives rise to an intriguing possibility at the interface of the attention/motivation and neuroimaging literatures; specifically, that the neurocognitive differences between approach and avoidance states may at least partially map on to these different neural sources of attentional flexibility. That is, it is possible that approach and avoidance motivational states facilitate attentional flexibility through different neural pathways, which in turn may help explain why approach leads to greater flexibility in some contexts and avoidance in others. Cognitive flexibility in approach states is believed to involve striatal dopamine [14], which is broadly consistent with conceptual and empirical links between approach motivation, reward responsivity, and striatal dopamine (e.g. [15, 16]). Additionally, sensitivity to reward has been linked to greater activity in the striatum as well as the inferior frontal cortex during task switching [17]. To our knowledge, however, the neural correlates of flexibility in avoidance states have not yet been studied in humans, and the role of striatal dopamine in avoidance states is less clear. Viewing aversive stimuli does not increase BOLD activity in the striatum [18]. On the other hand, studies in rats have shown that aversive stimuli do increase levels of striatal dopamine, which may reflect the motive to approach safety [19]. Thus, we would expect to see that activity in the striatum would be indicative of greater flexibility in approach states, but it is less clear whether similar regions would promote flexibility in avoidance states. The aim of the present study was to directly compare the neural correlates of attentional flexibility in approach and avoidance states across different contexts, with the goal of illuminating how these two opposing motivational states affect attention, and potentially longer-term outcomes.

## Materials and Methods

### Participants

Twenty-one participants were recruited from the University of Oregon community. Participants were right-handed, had normal or corrected-to-normal vision, no history of neurological or psychological disorders, and were free of MR contraindications (e.g., metal implants or claustrophobia). The task data reported here were collected as part of a larger study for which subjects were paid $60. Following data analysis, 2 subjects were excluded for having a low accuracy rate on the task (< 80%, all other subjects M accuracy = 96.77%), leaving 19 participants in the final analyses (10 female; M Age = 22.63, SD = 3.59, Range = 19–30).

#### Ethics Statement

All participants gave written informed consent to participate in the study. All study procedures were approved by the Committee for the Protection of Human Subjects at the University of Oregon.

### Procedure

The data reported here were collected as part of a larger two-session study. Each session included approximately 1.5 hours of scan time, and the task reported here was collected at either the first or the second session (but not both), counterbalanced across participants. This task was completed with two others involving inhibitory control (stop-signal) and emotion regulation (cognitive reappraisal), which are not relevant to present research questions and will not be discussed further. Participants provided informed consent upon arriving at the lab. After being situated in the scanner and performing the other tasks, participants were instructed on the task and completed 10 practice trials. Participants then completed 4 task runs. Following the scan, participants were asked several debriefing questions.

### Materials and Apparatus

#### Composite Letter Task with Motivation Manipulation

Participants completed a modified version of the composite letter task, in which the ratio of global to local targets changes across blocks of trials [5]. In this task, participants were presented with a large letter composed of several smaller letters (e.g. a T made of smaller Ls), and instructed to indicate with a button press whether the stimulus contained a T or an H. Each composite stimulus contained only one target letter (T or H), which was presented as either the large letter (global target) or the small letters (local target). Each trial had an equal probability of having a T or H target (though the probability of a global or local target was varied systematically by block as described below). Non-target letters, which constituted the other component of the composite letters, were L and F. Letter stimuli were white on a grey background. The height of the global letters subtended 3.37° of visual angle and the width 1.93°. The local letters had a height of 0.48° and a width of 0.32°. Stimuli were presented on a screen with dimensions 27.94 x 20.96 cm, and the viewing distance was 58.42 cm.

Before the presentation of each composite stimulus, participants viewed images intended to induce approach, avoidance, or neutral motivational states. Motivational state was manipulated by block. On approach blocks, images were of appetitive energy-dense foods (e.g., ice cream), the avoidance images were of insects and rotting food, and the neutral images were of everyday objects (e.g., light switch, filing cabinet). Each stimulus set consisted of 96 images, which were taken from stimulus sets used in previous studies [20,21] and the International Affective Picture System (IAPS; avoidance and neutral). The neutral stimulus set was supplemented with pictures from a Google image search. Approach (M = 3.91 on a five-point scale, SD = 0.12) and avoidance (M = 4.27, SD = 0.72) images were matched for intensity using pleasantness and unpleasantness ratings from previous studies using these stimuli. There was no significant difference in the intensity of pleasant versus unpleasantness ratings between the approach and avoidance images, t(97.7) = 1.32, p = .19 using a Greenhouse-Geisser correction. Each picture had a standardized width of 13.3° and a height that varied between 7.6–19.9° of visual angle.

Each trial began with a fixation cross presented for 1000 ms, followed by the motivation-inducing image for 750 ms. The fixation cross appeared again, for a randomly-jittered interval, with a gamma distribution and mean time of 500 ms. Next, the composite letter stimulus was presented for 1500 ms. Only responses provided within this 1500 ms window were included in the analyses.

Participants completed this task in blocks of 48 trials, and each block had one of three global-to-local ratios (i.e., contexts). Context was manipulated by block, as was motivation. On even blocks, there were an equal number of global and local targets, whereas the ratio was skewed on mostly global (36 global, 12 local) and mostly local (12 global, 36 local) blocks. In total, participants completed 9 blocks of this task, with one block for each cell in the Motivation (approach, avoidance, neutral) × Context (mostly global, even, mostly local) design. The blocks were distributed over 4 runs, 3 of which contained 2 blocks each, and 1 of which contained 3 blocks. After each block was completed, there was a 12-second rest period, during which the screen showed a fixation cross. The order of the runs was randomly varied across participants. The total task time was approximately 27 minutes.

#### Equipment

Stimuli were presented using eM’s Stimulus Software [22], which uses functions from the Psychtoolbox package in MATLAB. Participants made their responses on an MR-compatible button box using the middle and index fingers of their right hand.

#### Debriefing

Following their performance of the task, participants completed a funneled debriefing [23], to probe for awareness of the different block contexts by asking increasingly leading questions about how much they noticed. Participants were first asked “Did you notice anything about the blocks of trials?”. Next, they were asked whether they noticed “any differences between blocks of trials?”, and “that on some blocks of trials there was an uneven ratio of big letter to small letter targets?” Next, they were asked to guess how many, of the 9 experimental blocks, they believed had an uneven ratio of global to local trials. Finally, they were asked to guess the purpose of the experiment. In our previous behavioral work, relatively few subjects reported awareness of the ratio differences across blocks, and including subjects with awareness did not affect the behavioral findings.

### Behavioral Data Analysis

Prior to analysis, the behavioral data were cleaned by removing incorrect trials as well as those with reaction times (RTs) less than 100 ms, greater than 1500 ms, and also those that were greater than 3 SDs from each subject’s mean RT. RT data were analyzed using repeated measures ANOVAs.

### fMRI Data Acquisition

Data were collected on a 3T Siemens Allegra MRI scanner at the University of Oregon’s Robert and Beverly Lewis Center for Neuroimaging. The task described here involved 4 functional runs of T2*-weighted blood oxygenation level dependent echo-planar images (BOLD-EPI; TR = 2000 ms; TE = 30 ms; flip angle = 80°; matrix size = 64 x 64; 32 axial slices with interleaved acquisition; slice thickness = 4mm; field of view = 200 mm; in-plane resolution = 3.125 x 3.125 mm; bandwidth = 2605 Hz/pixel). Three of the runs (with two blocks each) contained 195 images and the other (with three blocks) contained 285 images. Motion was corrected in real time during functional runs with prospective acquisition correction (PACE).

High-resolution structural images were also acquired for each participant using a T1-weighted 3D MP-RAGE sequence that was coplanar with the functional images (TR = 2500 ms; TE = 4.38 ms; flip angle = 8°, matrix size = 256 x 192; 160 contiguous axial slices; voxel size = 1 mm^3^; slice thickness = 1 mm; bandwidth = 130 Hz/pixel). Additionally, field map scans were acquired for each participant to correct for field inhomogeneities (TR = 500 ms; TE = 4.99 ms; flip angle = 55°; matrix size = 64 x 64; field of view = 200 mm; 32 axial slices with interleaved acquisition; slice thickness = 4 mm, bandwidth = 1530 Hz/pixel).

### fMRI Data Analysis

First, data were skull-stripped using the Brain Extraction Tool in FSL (Functional Magnetic Imaging of the Brain Software Library). Data were then preprocessed and analyzed using SPM12b (Wellcome Department of Cognitive Neurology, London, UK). The preprocessing stream involved unwarping the functional images using the field maps, realigning the functional images to adjust for head motion, coregistering the functional images to each subject’s structural scan, manually reorientating to the anterior-posterior commissure line, normalizing to MNI space using DARTEL procedures, and smoothing using a 6-mm Gaussian kernel, FWHM.

At the single-subject level, three different types of event-related general linear models (GLMs) were used to analyze neural activity. Two types of models were run to model activity in the preparatory period, and the other type was used to examine activity during the response period. We ran these models separately so we could investigate the effect of context and motivation on neural activity during both the preparatory and trial periods while avoiding the high degree of multicollinearity between those adjacent periods of the task. All models included a first-order autoregressive error structure and within-subject global normalization to account for low-frequency drift. Additionally, in all models, the 6 motion parameters from the realignment step and brain activity during the 3 types of motivational image stimuli (approach, avoidance, neutral) were modeled as regressors of no interest.

For the preparatory period analyses, the jittered period between the offset of the motivational image stimuli and the onset of the composite letter stimuli (M duration = 500 ms) was modeled. The first analysis was modeled as a Motivation (Approach, Avoidance, Neutral) × Context (Mostly Global, Mostly Local, Even) factorial design with 9 regressors. Because subjects were unaware of the Target Type and whether there would be a switch on the upcoming trial, these factors were not of interest in this model. Four regressors modeling the trial period defined by the Switch (Yes, No) × Target Type (Global, Local) factorial structure were entered as regressors of no interest. These regressors were chosen to account for as much trial period variance as possible while minimizing their multicollinearity with the 9 preparatory period regressors. For the second preparatory period analysis, RT on the subsequent trial was entered as a parametric modulator of the preparatory brain activity, to allow us to identify the brain regions that predicted subsequent performance on different types of trials. Here, activity was modeled as a Motivation × Context × Switch factorial design with 18 regressors. We omitted the target type factor in this model because of insufficient power to examine the full 4-way interaction, and because we were particularly interested in neural activation in advance of switch and non-switch trials separately. Two regressors modeling the trial period (divided by target type) were entered as regressors of no interest. Linear contrasts were used to examine differences during the preparatory period between the conditions, and also how activity in the preparatory period was correlated with RTs.

For the response period models, the duration from stimulus onset to subject response was of interest. There was insufficient power to examine the 4-way interaction between all of the factors, so we ran a series of 3 models with the goal of examining all possible 3-way interactions involving the Motivation factor. For example, in one model, regressors were pooled across Target Type, leading to 18 regressors of interest: Motivation (approach, avoidance, neutral) × Context (mostly global, mostly local, even) × Switch (yes, no). The other two models pooled activity across Switch and Context respectively. These models included the preparatory period in the implicit baseline. Linear contrasts delineated the main effects of each factor, as well as all 2- and 3-way interaction effects.

For the group-level analyses, the single-subject contrast images were entered into single-sample t-tests with random effects. Monte Carlo simulations conducted using AFNI’s 3dClustSim determined that the minimum cluster size needed to maintain false discovery rate (FDR) of .05 with a voxel-wise threshold of p = .005 is 51. When necessary, parameter estimates for the clusters that emerged as significant were extracted using MarsBaR for visualization.

Our analytic approach for the neuroimaging data is as follows. There are four factors of interest (Motivation, Switch, Context, Target Type), two of which (Motivation and Context) have 3 levels. For these 3-level factors, we included only two of the levels in contrasts: for Motivation, contrasts focused on the comparison between approach and avoidance, whereas for Context, contrasts focused on the comparison between mostly global and mostly local conditions. We did this for simplicity, and because neutral motivation and even contexts were not central to our research goals.

The main research questions concern the effects of motivation and context on attentional flexibility (operationalized here as switching), thus we organize the presentation of the results to highlight the contrasts that are most relevant to these questions. Specifically, we will discuss the main effect of Switch first, then the two-way interactions of Switch × Motivation and Switch × Context, and finally the three-way interaction between Switch × Motivation × Context. These contrasts identify the neural correlates of attentional flexibility, as well as how they differ across different contexts and motivational states. For completeness and posterity, we conclude by presenting the other main effects and two- and three-way interactions, though they are not of primary interest here. Where there were significant interaction effects, parameter estimates were extracted from the entire cluster(s) within the relevant conditions for the purposes of plotting and interpretation.

## Results

### Debriefing Results

Subjects’ responses to the debriefing questions were examined, in order to determine whether results could be driven by subjects’ conscious awareness of the changing Global-Local ratio. No subjects answered yes to the first two debriefing questions (*Did you notice anything about the blocks of trials*? *Did you notice any differences between the blocks of trials*?*)*. Seven subjects answered yes in response to the third question (*Did you notice that on some blocks there was an uneven ratio of big letter to small letter targets*?). Of those subjects who answered yes to this third question, 6 answered the following question (*If you had to guess*, *how many of the 9 blocks had an uneven ratio of global to local targets*?), Mean = 5.33, SD = 1.97, Range = 4–9. In response to the final question, no subjects correctly guessed the purpose of the experiment. Given that all subjects who indicated noticing the ratio changes did so only after a fairly leading question, it is unlikely that subjects adopted a conscious task strategy in response to the changing global-local ratios. Furthermore, the percentage of subjects who noticed (37%) was similar to the percentage from the original behavioral study [5] (32%), and in that study, excluding the noticing subjects from analyses did not alter the results.

### Behavioral Results

Subjects’ mean reaction times (RTs) were examined using a Motivation: (Approach, Avoidance, Neutral) × Context (Mostly Global, Even, Mostly Local) × Switch (Switch, Non-Switch) repeated measures ANOVA. In cases where the assumption of equal variances is not met, the Greenhouse Geisser degrees of freedom are used. Switch trials were significantly slower than non-switch trials, *F*(1,18) = 78.81, *p* < .001, $\eta_{p}^{2}$ = .81. The main effects of Motivation and Context were not significant, *F*(1.21,21.79) = .29, *n*.*s*., $\eta_{p}^{2}$ = .02 and *F*(1.47,26.37) = .70, *n*.*s*., $\eta_{p}^{2}$ = .04, respectively. All of the 2- and 3-way interactions were tested. The interaction between Motivation and Switch was significant, *F*(2,36) = 5.87, *p* = .006, $\eta_{p}^{2}$ = .25. Post-hoc contrasts revealed that this interaction was driven by differences between the neutral and motivated (Approach and Avoidance) conditions, with the neutral condition leading to faster non-switch RTs but slower switch RTs compared to motivated states, *F*(1,18) = 11.84, *p* = .003, $\eta_{p}^{2}$ = .40. There were no differences between the Approach and Avoidance conditions on Switch versus Non-Switch RTs, *F*(1,18) = .46, *n*.*s*., $\eta_{p}^{2}$ = .025. Additionally, the interaction between Switch and Context approached significance, F(1.50,26.98) = 3.63, p = .052, $\eta_{p}^{2}$ = .17. Post-hoc contrasts revealed that this effect characterizes a pattern of Non-Switch trials having greater RTs on Even blocks and smaller RTs on Uneven blocks (Mostly Global, Mostly Local), and the opposite pattern for Switch trials, F(1,18) = 11.85, p = .003, $\eta_{p}^{2}$ = .40. All other 2- and 3-way interactions were not significant, *F*s < .50. Descriptive statistics for the behavioral results can be found in Table 1.

**Table 1:** Descriptive statistics for Switch x Motivation x Context ANOVA.

	Motivation			
		Approach	Neutral	Avoidance
**Context**	**Mostly Global**	**NS:** 648.2 (82.9)	**NS:** 617.4 (82.9)	**NS:** 639.9 (93.5)
		**SW:** 713.8 (120.0)	**SW:** 725.5 (120.6)	**SW:** 700.2 (112.1)
	**Even**	**NS:** 684.3 (96.4)	**NS:** 639.9 (183.4)	**NS:** 683.1 (88.0)
		**SW:** 714.7 (102.6)	**SW:** 698.0 (197.8)	**SW:** 720.2 (105.1)
	**Mostly Local**	**NS:** 609.8 (171.7)	**NS:** 677.6 (104.7)	**NS:** 643.5 (116.2)
		**SW:** 686.5 (194.2)	**SW:** 759.6 (123.1)	**SW:** 698.3 (105.6)

Mean and (Standard Deviation) of reaction time data in milliseconds. NS = Non-Switch, SW = Switch.

A separate ANOVA examined Context, Motivation, and Target Type, with the aim of determining the main and interacting effects of Target Type, which could not be included in the above analysis. There was no main effect of Target Type on RTs. However, Target Type did interact with Context in an expected manner (*F*(1.33,23.88 = 85.18, *p* < .001, $\eta_{p}^{2}$, = .83), such that there were faster RTs to global (vs. local) targets in mostly global contexts (*F*(1,18) = 29.87, *p* < .001., $\eta_{p}^{2}$ = .62) and faster RTs to local (vs. global) targets in mostly local contexts (*F*(1,18) = 64.75, *p* < .001., $\eta_{p}^{2}$ = .78). The difference in RT for global and local trials on even blocks did not reach significance (*F*(1,18) = 2.07, *n*.*s*., $\eta_{p}^{2}$ = .10). There was also a significant 3-way interaction between Target Type, Context, and Motivation (F(4,72) = 3.29, *p* = .02, $\eta_{p}^{2}$ = .15). Upon further examination with post-hoc contrasts, this interaction was driven by two significant effects. There were slower RTs for Global targets on Mostly Local blocks in Approach and Neutral versus Avoidance, *F*(1,18) = 11.29, *p* = .003, $\eta_{p}^{2}$ = .39. There were also slower RTs for Local targets in Mostly Global blocks in Neutral versus Approach and Avoidance Conditions, *F*(1,18) = 5.63, *p* = .029, $\eta_{p}^{2}$ = .24. Overall, this interaction suggests that the Neutral (and sometimes Approach) conditions led to slower responding to the non-dominant target types within a block. With the exception of the finding that RTs were faster when targets and contexts were congruent, these results do not replicate our behavioral findings. These null effects may have occurred because of a low N here (19) relative to our N in the behavioral studies (ranging from 42–46; totaling 131) or because the sensory differences between the scanning and behavioral testing environments (e.g., noise, light levels, distraction) may have obscured a relatively delicate behavioral effect. Nonetheless, the brain may still have the potential to reveal important information about how motivation and context affect the neural activity associated with attentional flexibility.

### Neuroimaging Results

#### Preparatory Period

Brain activity in the period immediately prior to stimulus presentation was analyzed to interrogate how context and motivation influence neural activity during preparation. The global > local context contrast revealed significant clusters in bilateral anterior cingulate cortex (ACC; k = 74, x = -3, y = 54, z = 12) and in the left posterior cingulate cortex (PCC; k = 70, x = -3, y = -48, z = 12). For the main effect of motivation, three cerebellar clusters emerged as more active in Avoidance compared to Approach trials (k = 65, x = 36, y = -57, z = -36; k = 58, x = 9, y = -54, z = -39; k = 63, x = -27, y = -45, z = -36. No significant clusters emerged for the interaction between Motivation and Context.

#### Preparatory Period with RT as a Parametric Modulator

**Fig 1:**
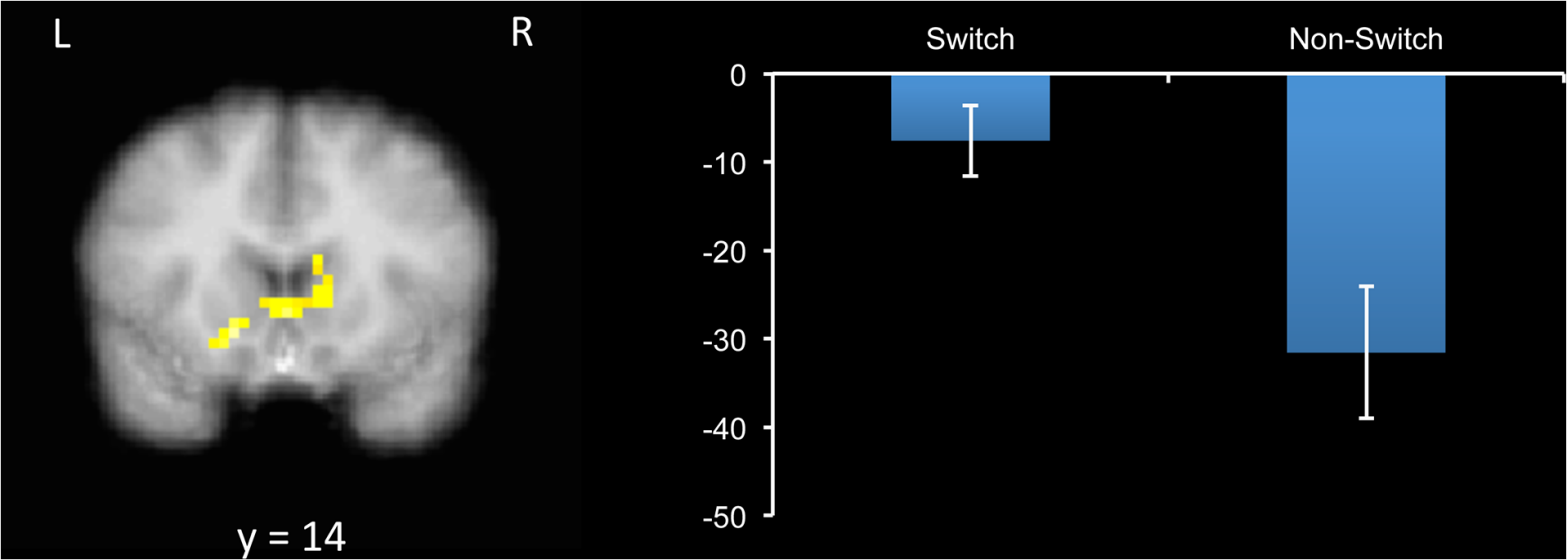
Cluster in bilateral dorsal striatum (DS), which was differently correlated with reaction time (RT) for switch and non-switch trials during the preparatory period. Activity in DS preceding the trial predicted faster RTs to a greater extent on non-switch trials. Graph shows parameter estimates, with the y-axis indicating the strength of the correlation between BOLD activity in this region and RT. Error bars represent +/- 1 standard error (SE).

Next, we entered RT as a parametric modulator of preparatory period activity to identify brain regions in which activity was correlated with task performance on the upcoming trial (Table 2). Here, the aim was to determine which brain regions’ activity was correlated with attentional flexibility, and whether the correlates of flexibility differ depending on motivation and context. A significant cluster in bilateral DS emerged as being correlated to a greater degree with RT in non-switch (vs. switch) trials (Fig 1). Parameter estimates were extracted, and they revealed that there was a negative relationship between RT and brain activity in this region on both switch and non-switch trials; however the relationship was stronger for non-switch trials. In other words, increased DS activity in preparation for a trial predicted faster responses on the trial, and particularly in non-switch trials.

**Table 2:** Key contrasts for the preparatory period analysis with RT as a parametric modulator.

Contrast	Laterality	k	x	y	z
***ME*: *Switch***					
Dorsal striatum (DS)	Bilateral	85	-15	24	-15
***2-way*: *Switch x Motivation***					
Anterior insula (AI)	Left	51	-36	21	0
***2-way*: *Switch x Context***					
—					
***3-way*: *Switch x Motivation x Context***					
Superior temporal gyrus (STG)	Right	75	63	-21	15
Inferior parietal lobule (IPL)	Right	64	42	-39	51

Clusters reached significance if they surpassed the voxel-wise significance threshold of p < .005 and contained more than 51 voxels, to achieve a FDR of .05. ME = main effect, k = cluster size (in voxels), x,y,z represent the location of each cluster’s peak activity, in MNI coordinates.

**Fig 2:**
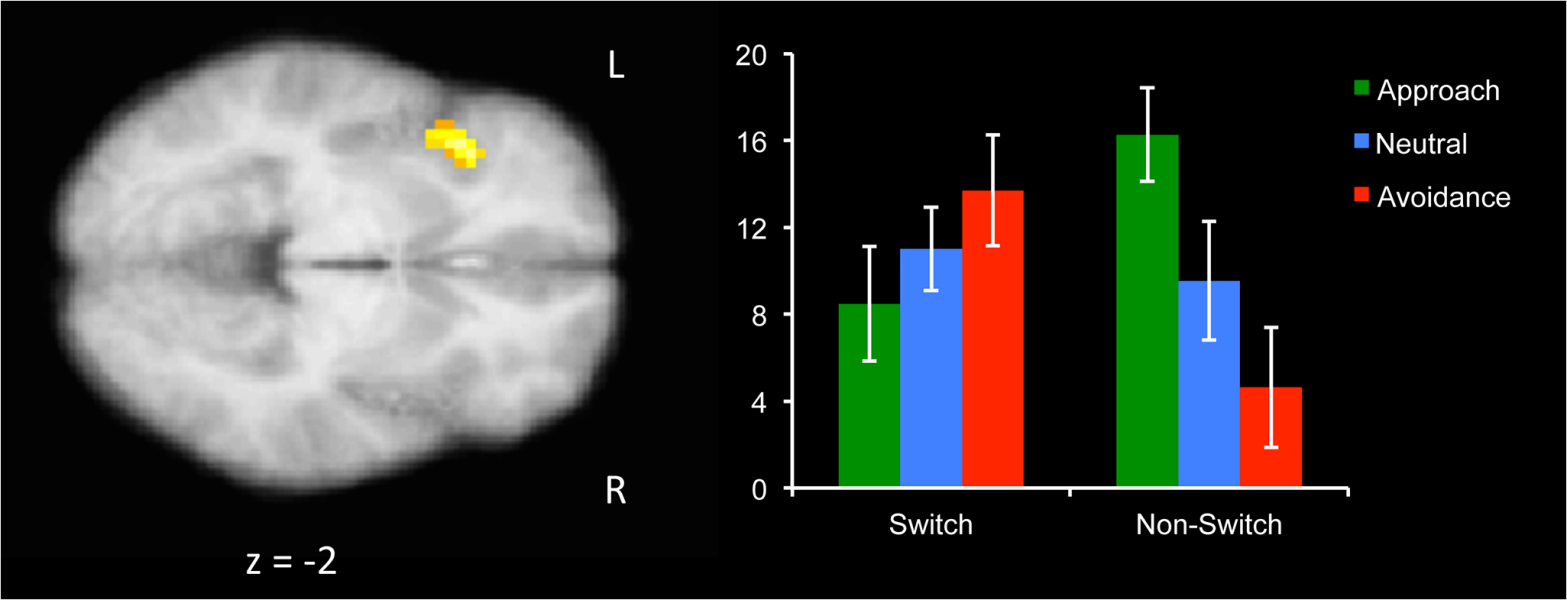
Cluster in left anterior insula (AI), which was differently correlated with RT, depending on Switch (switch, non-switch) and motivation (approach, avoidance) during the preparatory period. Activity here predicted slower responding, especially on avoidance-switch trials and approach-non-switch trials. Graph shows parameter estimates, with the y-axis indicating the strength of the correlation between BOLD activity in this region and RT. Error bars represent +/- 1 SE.

The next step was to determine whether the neural correlates of attentional flexibility change as a function of motivation or context. There was a 2-way interaction between Motivation and Switch in a cluster in the left anterior insula (AI; Fig 2; Table 2). On switch trials, avoidance motivation had a stronger positive relationship between RT and activity in this insula region, whereas on non-switch trials, the relationship was stronger for approach motivation. That is, activity in the left AI predicted slower responding on switch trials when individuals were in an avoidant state, but slower responding on non-switch trials when individuals were in an approach state. No regions reached the threshold for the interaction between Switch and Context.

**Fig 3:**
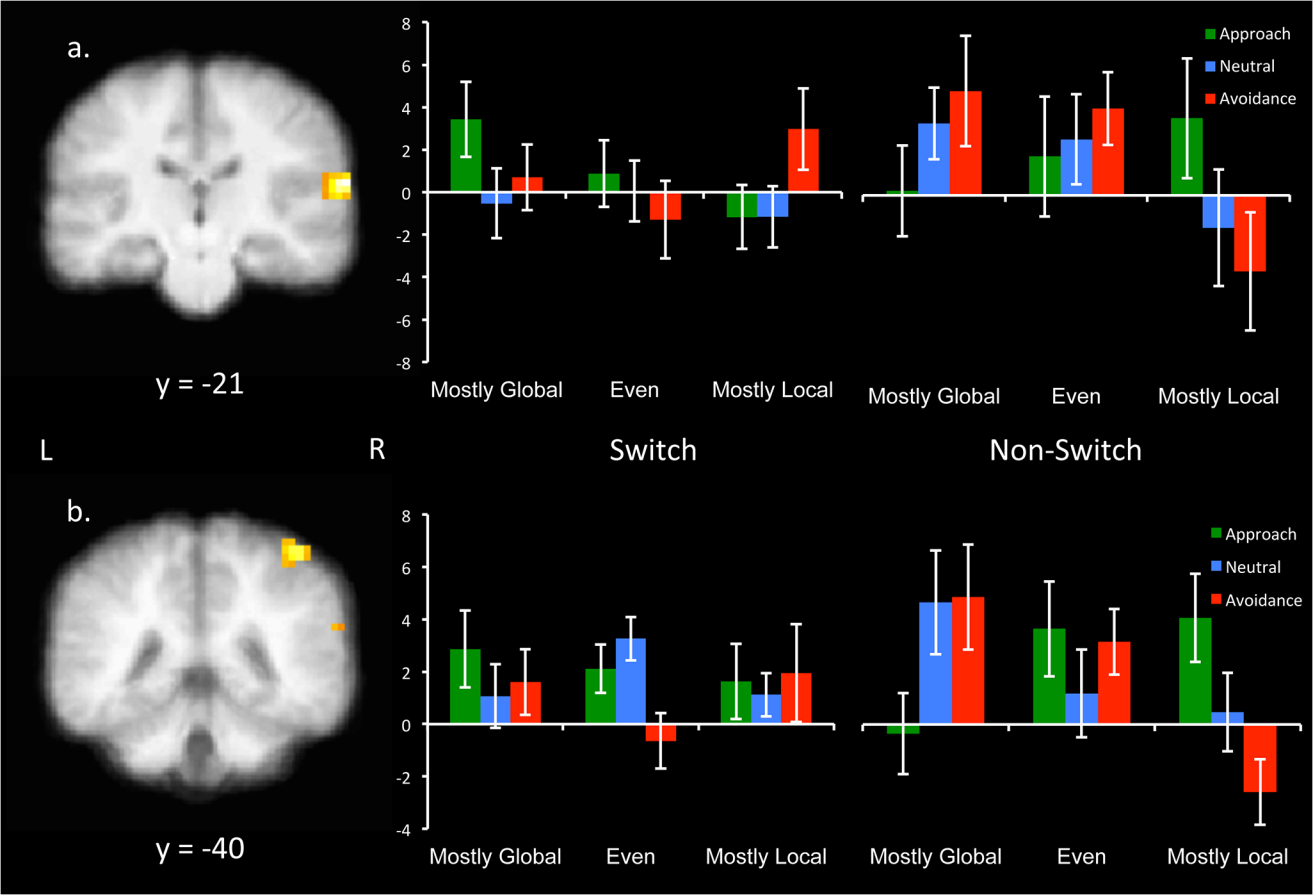
Preparatory activity in (A) right superior temporal gyrus (STG) and (B) inferior parietal lobe (IPL) was differently correlated with RT depending on both Switch (switch, non-switch) Motivation (Approach, Avoidance), and Context (mostly global, mostly local). Graph shows parameter estimates, with the y-axis indicating the strength of the correlation between BOLD activity in this region and RT. Error bars represent +/- 1 SE.

Finally, the RT-brain activity relationships were examined for the 3-way interaction between Switch, Motivation, and Context. Here, there were significant clusters in right superior temporal gyrus (STG) and right inferior parietal lobule (IPL; Table 2). As shown in Fig 3A, activity in rSTG predicted slower RTs when switching in mostly global approach blocks and mostly local avoidance blocks. For non-switch trials, this relationship was reversed, with activity predicting slower RTs in mostly global avoidance blocks and mostly local approach blocks. The IPL cluster had a similar pattern of results (Fig 3B). Thus, there were brain regions in which the relationship between attentional flexibility performance and preparatory activity differed across different motivational states and contexts.

In addition to the contrasts that were driven by the research questions, other contrasts examined the remaining main effects and interactions (Table 3). There was a main effect of Context, in which activity in five regions including the cingulate cortex and the middle frontal gyrus had a strong negative relationship with RT in mostly global contexts, but not in mostly local contexts. One explanation for this finding is that activity in these regions are involved in processing the global stimuli, and thus activity here would correlate with faster RTs to an increasing degree as the proportion of global targets increases. No regions reached threshold for the main effect of Motivation or for the Motivation × Context interaction.

**Table 3:** Other contrasts for the preparatory period analysis with RT as a parametric modulator.

Contrast	Laterality	k	x	y	z
***ME*: *Context***					
Cingulate gyrus	Left	122	-12	-3	33
Middle frontal gyrus (MFG)	Right	96	24	42	-12
Thalamus/pulvinar	Bilateral	51	-12	-30	12
	Right	144	18	-39	9
Middle frontal gyrus / precentral gyrus	Right	90	27	3	30
***ME*: *Motivation***					
—					
***2-way*: *Motivation x Context***					
—					

Clusters reached significance if they surpassed the voxel-wise significance threshold of p < .005 and contained more than 51 voxels, to achieve a FDR of .05. ME = main effect, k = cluster size (in voxels), x,y,z represent the location of each cluster’s peak activity, in MNI coordinates.

#### Response Period

Next, we examined brain activity during the response period—when participants searched the composite stimuli for the target letters. For the main effect of Switch (Table 4), a region in the left IPL showed greater activity on switch (vs. non-switch) trials, whereas a region in left middle temporal gyrus (MTG) showed greater activity on non-switch (vs. switch) trials.

**Table 4:** Key contrasts for the response period analysis.

Contrast	Laterality	k	x	y	z
***ME*: *Switch > Non-Switch***					
Inferior parietal lobule (IPL)	Left	64	-39	-42	36
***ME*: *Non-Switch > Switch***					
Middle temporal gyrus (MTG)	Left	67	-42	-75	21
***2-way*: *Motivation x Switch***					
Anterior cingulate cortex (ACC)	Bilateral	60	-6	54	0
***2-way*: *Switch x Context***					
Occipital lobule	Left	100	-18	-99	-3
***3-way*: *Switch x Motivation x Context***					
—					

Clusters reached significance if they surpassed the voxel-wise significance threshold of p < .005 and contained more than 51 voxels, to achieve a FDR of .05. ME = main effect, k = cluster size (in voxels), x,y,z represent the location of each cluster’s peak activity, in MNI coordinates.

**Fig 4:**
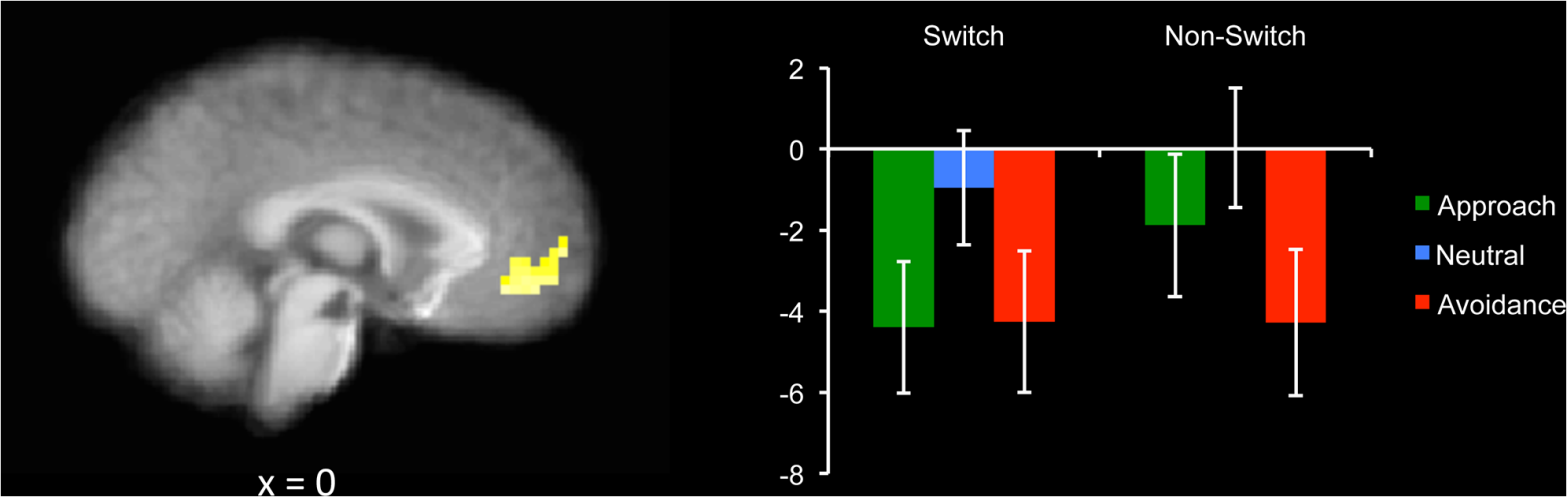
Response period activity in bilateral anterior cingulate cortex (ACC), which showed an interaction between Switch (switch, non-switch) and Motivation (approach, avoidance). There tended to be less ACC activity across both approach and avoidance and switch and non-switch conditions compared to baseline; however the reduction in activity was less for approach-non-switch trials. Graph shows parameter estimates, with the y-axis indicating the magnitude of BOLD activity in arbitrary units. Error bars represent +/- 1 SE.

We then examined the key interactions involving motivation (Table 4). A cluster within the bilateral ACC emerged in the interaction between Motivation and Switch (Fig 4). In this region, both approach and avoidance showed reduced activity during switch trials; however during non-switch trials, activity was reduced to a lesser degree in the approach condition. A region in the left occipital lobe emerged in the Switch × Context interaction (Table 4). There, both switch and non-switch trials showed increased activity in mostly global blocks, whereas only switch trials showed increased activity in mostly local blocks. No significant clusters emerged from the 3-way interaction between Motivation, Switch, and Context.

For the sake of completeness, the other main and interaction effects were investigated (Table 5). The main effect of Context contrast revealed significant clusters in several regions. There was greater activity in right PCC in global (vs. local) contexts, whereas several regions, including the rSTG, the left inferior frontal gyrus (IFG), and the left cingulate were more active in local (vs. global) contexts. For the Target Type factor, participants on average had significantly greater activity in left middle occipital gyrus when responding to global (vs. local) targets. No regions showed a main effect of Motivation during the response period.

**Table 5:** Other contrasts for the response period analysis.

Contrast	Laterality	k	x	y	z
***ME*: *Mostly Global > Mostly Local***					
Posterior cingulate cortex (PCC)	Right	68	3	-39	24
***ME*: *Mostly Local > Mostly Global***					
Superior temporal gyrus (STG)	Right	155	36	-48	21
Cingulate gyrus	Left	401	-18	-42	24
Fusiform gyrus / lingual gyrus	Left	126	-33	-45	3
Frontal; near claustrum	Right	193	27	15	18
Inferior frontal gyrus (IFG)	Left	98	-36	39	0
***ME*: *Global Target > Local Target***					
Occipital lobule	Left	308	-21	-93	0
***ME*: *Local Target > Global Target***					
—					
***ME*: *Approach >/< Avoidance***					
—					
***2-way*: *Motivation x Context***					
Medial parietal cortex / Precuneus	Left	67	-24	-57	24
***2-way*: *Context x Target Type***					
Superior temporal lobule (STL)	Left	138	-51	-57	12
Cingulate gyrus	Left	100	-6	-42	45
Inferior parietal lobe (IPL)	Left	298	-39	-48	45
Precentral gyrus	Left	84	-48	3	24
Precuneus	Right	52	24	-63	42
***2-way*: *Switch x Target Type***					
—					
***3-way*: *Motivation x Switch x Target Type***					
Inferior temporal gyrus (ITG)	Right	94	51	-60	-6
Superior temporal gyrus (STG)	Right	85	48	-30	12
***3-way*: *Motivation x Context x Target Type***					
—					

Clusters reached significance if they surpassed the voxel-wise significance threshold of p < .005 and contained more than 51 voxels, to achieve a FDR of .05. ME = main effect, k = cluster size (in voxels), x,y,z represent the location of each cluster’s peak activity, in MNI coordinates.

The interaction between Motivation and Context revealed a cluster in the left parietal lobe, in which activity on mostly global trials was increased during approach and decreased during avoidance, and showed the opposite pattern for mostly local blocks. Additionally, several regions emerged in the interaction between Context and Target Type. In left superior temporal gyrus (lSTG) and left cingulate cortex, there was relatively greater activity for targets that were more frequent in the context (e.g., global targets in mostly global trials). On the other hand, in the left inferior parietal lobe (lIPL), left precentral gyrus, and right precuneus the pattern was the opposite, with greater activity for targets that were less frequent in the context (e.g. local targets in mostly global blocks). No regions emerged for the Switch × Target Type interaction.

Finally, significant clusters emerged in the right inferior temporal gyrus (rITG) and rSTG in the 3-way interaction between Motivation, Switch, and Target Type. Activity in rITG was greater for approach-global targets and avoidance-local targets on switch trials, but had the reversed pattern on non-switch trials, with greater activity on approach-local trials and avoidance-non-switch trials. On the other hand, activity in rSTG showed the opposite pattern. No other regions evinced a 3-way interaction.

## Discussion

The goal of the present study was to examine how approach and avoidance motivational states affect the neural mechanisms involved in attentional flexibility. To this end, the experimental task required participants to shift their focus between attending global and local features of a stimulus while in approach and avoidance states. Faster switching was indicative of greater flexibility. Because previous research demonstrated that some differences between motivational states emerge only across different contexts [5], the ratio of global to local stimuli was also varied across blocks, in order to maximize the possibility of observing motivational differences.

The results demonstrated that there are some clear differences between approach and avoidance states in the neural mechanisms that support attentional flexibility. This finding is broadly consistent with earlier work showing four separate networks for attentional flexibility in humans [13], although the specific brain regions observed here were different. In terms of preparedness to shift attention, a region in the left AI was activated differently in approach and avoidance states. Here, pre-trial activity predicted slower responding on switch trials for those in avoidance states; whereas it predicted slower responding on non-switch trials when subjects were in an approach state. Furthermore, pre-trial activity in right STG and right IPL predicted performance differences based on both motivation and task context. Greater activity in those regions predicted slower switching under approach-mostly global and avoidance-mostly local conditions. On non-switch trials, the relationship was reversed, with greater activity predicting slower responding on approach-mostly local and avoidance-mostly global blocks. Together, these findings implicate specific brain regions that may be involved in motivation-dependent modulation of preparedness to switch between different perceptual dimensions. This finding builds on the work by Leber and colleagues [13] by beginning to delineate context-specific roles for the distinct networks involved in shifting.

We also examined brain regions involved in the actual process of shifting attention, and how they differed across motivational states. In this analysis, a region in the bilateral ACC emerged as having different flexibility-related activity between approach and avoidance conditions. In this region, differences between approach and avoidance emerged on non-switch trials, with approach leading to a smaller decrease in activity during this period than avoidance.

These findings are noteworthy because they demonstrate that approach and avoidance cause a shift in the underlying brain activity during an attentional flexibility task in spite of the fact that those motivational states can, in some cases, have similar behavioral effects on attention [24,25]. These neural differences may reflect different pathways through which approach and avoidance support attentional flexibility, and in particular the ability to flexibly shift attention between perceptual dimensions. The present research does not speak to the question of whether approach or avoidance affords greater flexibility; however, it does suggest that flexibility is supported via different neural mechanisms, depending jointly on one’s motivational state and the task context.

This study was the first to investigate the interacting effects of motivation, context, and attentional switching on neural activity, so it is difficult to compare its results to previous research. Nonetheless, several results emerged here that are interesting to consider in light of earlier work. First, the preparatory period analysis demonstrated that activity in regions in left AI, as well as right STG and IPL differed with respect to their relationship to switching across motivation conditions (and context, in the case of the latter two regions). In the case of the AI, pre-trial activity predicted slower responding on both switch and non-switch trials; however the slowing was greatest for avoidance-switch and approach-non-switch trials. In the Leber et al. [13] study, preparatory activity in the right AI was correlated with faster switching on an upcoming trial, which contradicts our findings here. On the other hand, they did not find significant activity in the left AI, so it may not be useful to compare findings across these two studies. Recently the insula, along with the ACC, has been proposed as a hub for a salience network, which detects significant events in the environment and redirects cognitive resources in order to deal with them [26]. The role of the insula as a salience detector fits well with the current data. The preparatory activity modeled here reflects the brain directly following viewing motivationally relevant images. Both approach and avoidance images would have been highly salient, but interestingly, AI activity predicted less flexibility following avoidance images and greater flexibility following approach images. Thus, a potential interpretation of this finding is that the salience network is sensitive to differences between approach and avoidance states.

Preparatory activity in the right IPL, which emerged in the present study, has been found to correlate with greater attentional flexibility in previous research [13]; however in our study, preparatory activity in this region predicted slower switching on most types of switch trials, albeit to differing degrees. The relationship between right IPL activity and switch costs was especially pronounced on approach trials in mostly global blocks and avoidance trials in mostly local blocks. Right STG showed a similar pattern of activity; however STG has not been as closely linked to flexibility in previous studies.

The main finding in the response period analysis, that activity in ACC interacted with both motivation and switch also did not necessarily agree with previous findings. Both approach and avoidance motivation led to reduced activity here, compared to neutral, on both switch and non-switch trials. Previous research has suggested that the ACC is involved in salience detection and conflict monitoring [13] as well as the allocation of cognitive control resources [27]. It is not clear here whether this ACC function relates to the present study, given that appearance of trial stimuli tended to reduce activity here compared to baseline. As will be discussed in more detail below, task differences between this and previous studies may help explain why there was not greater activity in the ACC during this study.

Another important difference between the findings of the present study and previous studies of cognitive flexibility is for the main effect of switch. Unlike some previous studies [12,13], we did not find a broad neural network of frontal, partietal, and subcortical regions involved in attentional flexibility. In the preparatory period analysis, there was a single significant cluster in the DS, whereas in the response period left IPL activity was greater on switch trials, and left MTG activity was greater on non-switch trials. Activity in left IPL occurred during switching, which agrees with previous research (e.g. [12]). IPL is also believed to be involved in bottom-up reorienting of attention [28], which may occur in the case of a switch to a new perceptual dimension. On the other hand, in the preparatory period analysis, activity in the bilateral DS was actually predictive of slower RTs, rather than faster RTs, which would have been expected from previous research demonstrating the role of the DS in facilitating switching on an upcoming trial [13]. Additionally, based on the findings of Aarts et al. [14], we had hypothesized that DS activity may be more predictive of flexibility in approach states compared to avoidance states; however, there was no evidence of greater approach-related DS activity in these data. As will be outlined below, there are several noteworthy differences between the implicit attentional shifting task used in the present study and more explicitly-cued switching tasks used in most previous studies. Additionally, it is important to note that Aarts et al. [14] used actual monetary rewards to induce approach motivation, whereas in the present study, approach motivation was induced by passive viewing of appetizing photos. It is likely that approach motivation that is directed towards attaining an actual reward is different from the more diffuse approach state that may come from viewing appetizing images. Nonetheless, the fact that the DS is consistently implicated in a variety of studies of attentional flexibility suggests that it plays a role in supporting attentional flexibility. The finding that the direction of its activity is sensitive to task differences is also interesting, and future research will be important to better understand exactly how the DS is involved in different types of attentional shifts.

There were also several key design differences between the present study and others, which may help explain these seemingly contradictory findings. First, the present study examined participants’ ability to shift attention between broad and narrow focus, rather than the ability to switch between using multiple task rules (e.g. [14, 13]). Unlike in these other studies, the rule in the present study remained the same throughout the task: Subjects responded about whether the stimulus contained a T or an H, and although the spatial properties of the target could change, the basic task rule did not. Furthermore, because the task rule did not change from trial to trial, there was no need for instructional cues before each trial. Most previous studies looking at switching have used cues, either preceding or simultaneous with test stimulus presentation. For example, in the study by Hedden and Gabrieli [12], the color of the composite letter stimulus informed participants whether they should attend and respond the global or local stimulus features. Although the Leber et al. study [13] examined pre-cue activity, it is still important to note that the task itself was cued. The lack of cues and rule changes in the present study meant that the shifts of attention were more subtle, and potentially occurred outside of subjects’ conscious awareness. Finally, several other studies of switching also require inhibition of some sort, to suppress either the urge to use an old task rule (e.g. [14]) or to attend a target in an irrelevant stimulus dimension (e.g. [12]). In the present study, however, demands on an inhibitory system would have been minimal, because the other letters that made up the composite stimuli were always non-target letters (e.g. L or F), and were never relevant to the task, thus it may have required little effort to ignore them. Many of the areas involved in switching overlap [12] or interact [29] with regions that are involved with inhibition, and thus reduced demands on an inhibitory system may have resulted in less robust activation for the main effect of switch. There are, therefore, several factors that may have contributed to the different findings between the present study and other studies. These task differences, paired with the unexpected findings in the present study, underscore the importance of careful consideration of the specific type of flexibility that is being examined in different studies.

One limitation of the present study is that there were few significant behavioral effects, thus, it is not possible to make connections between the observed neural differences and differences in behavior across conditions. Our previous research [5] with a larger sample did find context- and motivation-dependent changes in attentional flexibility between approach and avoidance states. It is unclear whether a larger sample would have led to significant behavioral results in the present study. On the other hand, the fact that neural differences emerged in the absence of behavioral differences suggests that the underlying neural differences in the mechanisms underlying flexibility may be more robust than behavioral effects. This pattern fits with our conceptual model that the observed neural changes are proximal determinants of downstream behavior, so any manipulation will have a weaker effect on behavior than on the closer neural systems. Nonetheless, the small sample size is certainly a limitation of the present study, as the low power may have undermined its ability to obtain significant results [30].

Another limitation of the study design is that some conditions, namely minority non-switch trials (e.g., local non-switch trials on mostly global blocks) had very few trials. Because of this necessity, it was not possible to analyze the interaction of all four factors (Switch, Motivation, Context, Trial Type) simultaneously. It will be possible to narrow the scope of future studies, thereby allowing for fewer conditions of interest, which could ameliorate the problem of having too few trials in a given condition.

Additionally, we do not have arousal ratings for all of the approach and avoidance stimuli. Although motivational intensity and arousal are often closely related, we cannot rule out the possibility that the different picture sets varied in the level of arousal they elicited.

Finally, it is important to note that the scanning environment may have limited our ability to reliably evoke strong motivational states, especially in the Approach condition. Lying down in a supine position, as required in the scanner, is not compatible with approach-motivated action tendencies. Indeed, a supine position leads to a reduction in approach-related neural activity, as measured by EEG [31], presumably via an embodiment mechanism. On the other hand, forward-leaning bodily postures, which are more consistent with approach action tendencies, tend to increase approach-related neural activity [32]. Unfortunately, there are no ways to remedy this situation given the current state of MRI hardware. It will be important to supplement fMRI findings with those using other neuroimaging methods that do not require the subject to lie down.

Overall, however, the present study suggests that the neural mechanisms supporting attentional flexibility differ across approach and avoidance motivational states, both during the preparatory period and during the response period. The specific type of switch examined here is novel relative to what has been done in other studies. Whereas other studies have examined explicit, cued task switching, the present study examines more subtle, implicit attentional shifts between global and local features of an object. Such a shift would support the ability to overcome one’s current level of attentional focus to find a sought-after object in the environment. The differences in neural activity that emerge between approach and avoidance states may have important implications for understanding behavior, both within a brief situation and over the longer term, in the case of individuals who tend to chronically experience one type of motivation.
